# Fluids in sepsis

**DOI:** 10.1016/j.bjae.2026.04.003

**Published:** 2026-06-29

**Authors:** R.R. McMullan, J.A. Silversides

**Affiliations:** 1 Queen’s University of Belfast, Belfast, UK; 2 Belfast Health and Social Care Trust, Belfast, UK

**Keywords:** deresuscitation, fluid therapy, oedema, resuscitation, sepsis

**Key points**

• Sepsis-induced hypotension is a multifaceted process driven by a dysregulated immune response.
• The revised Starling principle highlights the key role of the endothelial glycocalyx.
• There is an expansive body of evidence showing harm associated with i.v. fluid therapy in sepsis.
• Restrictive fluid strategies appear to be safe in selected patients with sepsis-induced hypotension.
• A fluid deresuscitation strategy may mitigate the detrimental effects of fluid overload.

**Learning objectives**

By reading this article, you should be able to:

• Describe the aetiology of sepsis-induced hypotension.
• Explain the key differences between the Starling principle and the revised Starling principle.
• Discuss the benefits and harms associated with i.v. fluid therapy in sepsis.
• Describe the role of fluid deresuscitation.

Sepsis is a state of organ dysfunction caused by a dysregulated host immune response to infection.^1^ Sepsis is associated with high morbidity and mortality, with an estimated 48,000 people dying from sepsis in the United Kingdom each year, with annual NHS costs of up to £2 billion.^2^

## Sepsis pathophysiology

The host response is triggered by pathogen-associated molecular patterns (PAMPs) and damage-associated molecular patterns (DAMPs). PAMPs are molecular motifs derived from microbes and include lipopolysaccharide, lipoproteins, peptidoglycans and lipoteichoic acid.^3^ DAMPs are derived from stressed or injured cells and include heat shock proteins, S100 proteins, hyaluronic acid degradation products and host deoxyribonucleic acid fragments.^3^ PAMPs and DAMPs are recognised by a variety of pattern recognition receptors (PRRs), such as toll-like receptors and C-type lectin receptors.^3^ Ligand binding to PRRs on host immune cells activates intracellular signalling cascades and cytokine generation.

Cytokines recruit immune cells, such as neutrophils, to the site of tissue injury. However, in sepsis, the dysregulated nature of the immune response results in excessive generation of potent pro-inflammatory cytokines, such as tumour necrosis factor-α and interleukin-6. Pro-inflammatory cytokines have been shown to mediate the breakdown of key endothelial cell adhesion molecules, which contributes to vascular leakage and the formation of interstitial oedema.^4^ After the initial hyperinflammatory phase, immunosuppression may occur through mechanisms including lymphocyte apoptosis and impaired autophagy, increasing the risk of secondary infection.^5^

### Sepsis-induced hypotension

Septic shock is defined in clinical practice by the need for vasopressor drugs to maintain a mean arterial pressure of ≥65 mmHg despite adequate resuscitation with i.v. fluids, and a serum lactate concentration >2 mmol L^−1^. The development of septic shock is a multifaceted process and a harbinger of poor clinical outcome, with a 40% mortality rate.^1^ However, patients with persistent sepsis-induced hypotension *without* hyperlactataemia are also encountered in clinical practice and generally have a lower mortality than those meeting the full definition for septic shock.

### Dysregulation of vasoactive pathways

Endothelial cells synthesise nitric oxide (NO), a key mediator of vascular smooth muscle relaxation. In pathological states, excessive NO production can contribute to profound vasoplegia. Various inflammatory mediators, including cytokines, have been implicated in the excessive and dysregulated activity of NO in sepsis.^6^ Moreover, sepsis results in the downregulation of key vasoconstrictive receptors, which can be observed in clinical practice as a state of vasopressor-refractory shock. Experimental models of sepsis have confirmed the downregulation of angiotensin II receptors and α_1_-adrenergic receptors. This is accompanied by downregulation of vasopressin V_1_ receptors and deficiency in circulating vasopressin concentrations, which has prompted the use of vasopressin and its analogues in septic shock.^7^

### Septic cardiomyopathy

Septic cardiomyopathy (SCM) is identified as a global but reversible dysfunction of both sides of the heart induced by sepsis. A key feature of SCM is its reversibility, with survivors recovering full cardiac function within 7–10 days. The reported prevalence ranges from 10% to 70%, although the epidemiology remains uncertain because there is no consensus definition. Risk factors for the development of SCM include male sex, younger age, increased lactate concentrations, and a history of heart failure.^8^ Echocardiogram findings typically reveal ventricular dilation with increased compliance and normal to low filling pressures. The left and/or right ventricle may be affected, and systolic dysfunction, diastolic dysfunction, or a mixed pattern may be observed.^8^

The pathogenesis of SCM is likely to be multifactorial. Pro-inflammatory cytokines directly depress myocardial cell contractility and trigger the release of other factors, such as NO, that can adversely affect myocardial contractility through the production of reactive oxygen species, altered calcium sensitivity, and catecholamine receptor downregulation.^8^

### Vascular leakage

**Fig 1:**
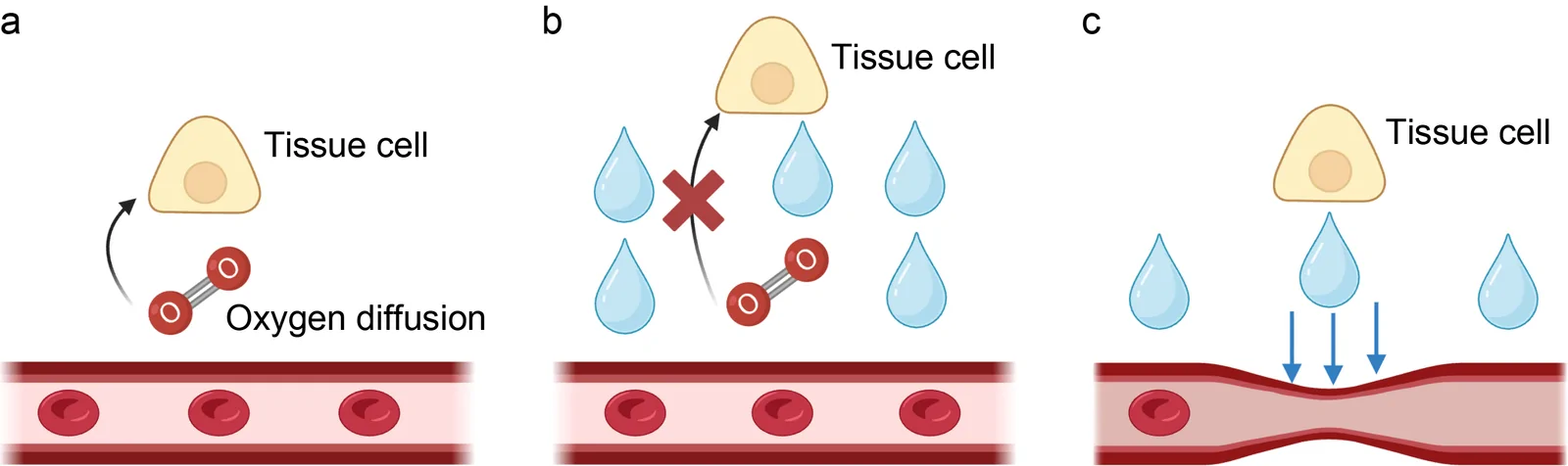
Effects of tissue oedema on oxygen delivery to cells. Reproduced from^4^ under Creative Commons license. a) Normal oxygen diffusion from blood vessels to target tissue cells. b) Impaired oxygen delivery because of increased diffusion distances and impaired oxygen solubility in interstitial fluid. c) Impaired oxygen delivery caused by extrinsic compression of the microvasculature resulting from the tamponade effects of interstitial fluid.

A hallmark feature of sepsis is endothelial and microcirculatory dysfunction.^4^ Injury to interendothelial junctional structures culminates in the loss of fluid from the intravascular space to the interstitial space. Tissue oedema leads to increased diffusion distances between capillaries and cells. Impaired oxygen delivery is further compounded by poor oxygen solubility in interstitial water (Fig. 1a and 1b). Furthermore, it is hypothesised that excessive infusions of i.v. fluids lead to an increase in venous pressure that is transmitted to the capillary circulation, where the increase in hydrostatic pressure leads to net extravasation of fluid and oedema formation. Microvascular blood flow may be further compromised by mechanical extrinsic compression of capillaries and lymphatics by interstitial fluid (Fig. 1c).^9^ Tissue hypoxia secondary to oedema may explain the adverse outcomes associated with fluid overload in patients with sepsis and other inflammatory conditions.

#### Starling principle

In the original form, the Starling principle explained the movement of fluid between the circulating plasma and the interstitium as being determined by hydrostatic pressure and colloid oncotic pressure gradients between plasma and interstitial fluid.

The classic Starling equation describes net transvascular fluid movement as:

Jv = Kf ([Pc − Pi] − σ[πc − πi])

where Jv is net fluid movement, Kf the filtration coefficient, Pc capillary hydrostatic pressure, Pi interstitial hydrostatic pressure, πc capillary oncotic pressure, πi interstitial oncotic pressure, and σ the reflection coefficient. It was considered that fluid moved from the plasma into the interstitial space at the arteriolar portion of capillaries, where the hydrostatic pressure gradient dominates, and fluid was absorbed from the interstitial space at the venular portion of capillaries because of the dominant ‘draw’ of plasma colloid oncotic pressure.^10^

#### Revised Starling principle

Over the years the Starling principle has evolved, and it is now accepted that while non-fenestrated capillaries filter fluid to the interstitium, the majority of filtered fluid returns to the circulation as lymph.^10^ The classical Starling principle assumed that interstitial oncotic pressure was significantly lower than capillary oncotic pressure; however, it is now recognised that interstitial oncotic pressure approaches that of capillary oncotic pressure.^11^ Both physiological extravasation of plasma proteins through capillary pores in health and pathological extravasation associated with endothelial injury result in a high interstitial oncotic pressure.

The revised Starling principle emphasises the importance of the endothelial glycocalyx, which regulates transvascular fluid exchange.^10^ The glycocalyx, a network of proteoglycans and glycoproteins, lines the luminal surface of vascular endothelium. Water and electrolytes pass freely across the endothelial glycocalyx and then beyond endothelial cells into the interstitial space via intercellular clefts, whereas larger molecules, such as albumin, are unable to pass through an intact glycocalyx and rely on active processes to be transported across endothelial cells.

**Fig 2:**
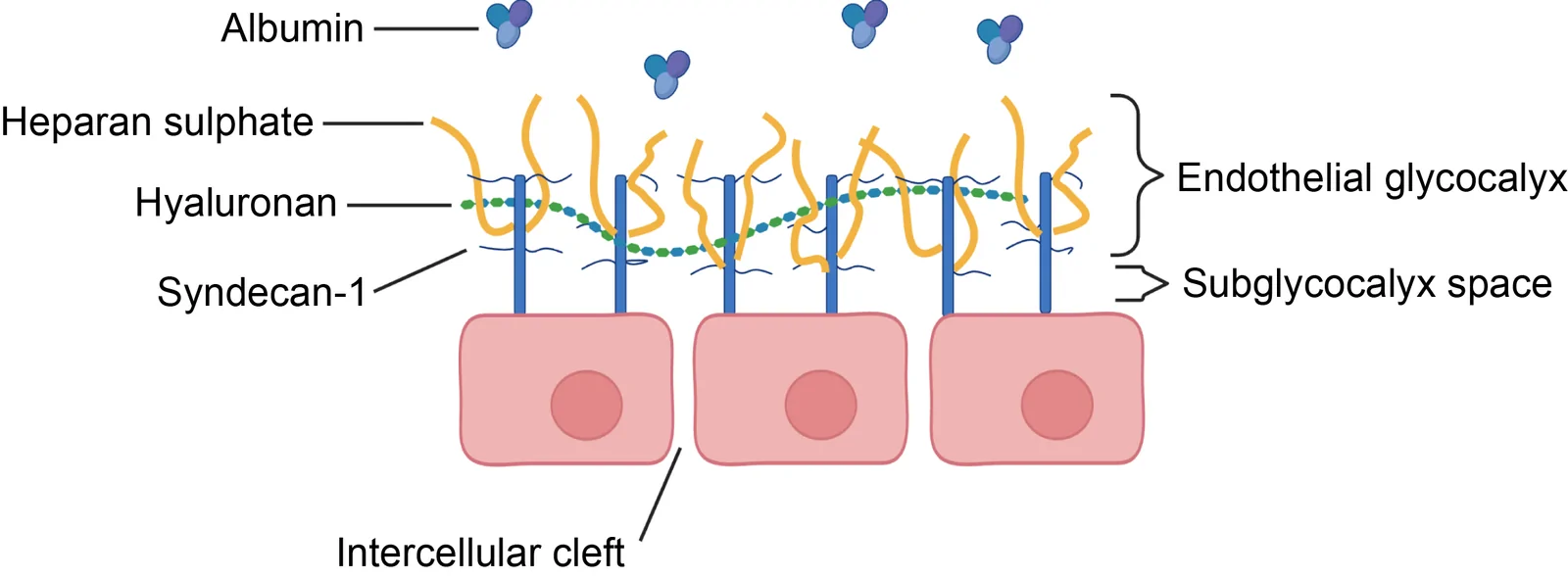
Fluid movement between the endothelial glycocalyx, subglycocalyx space, and endothelial cells. The subglycocalyx space is a protein-free zone between the glycocalyx and the endothelial cells. An oncotic gradient develops from the low protein concentration of the subglycocalyx space to the intravascular space, and this serves to oppose outward fluid filtration.

Just below the glycocalyx layer is a region referred to as the subglycocalyx space, which is almost protein-free (Fig. 2). The colloid oncotic pressure of the subglycocalyx space replaces the colloid oncotic pressure of the interstitium as a determinant of transvascular fluid movement.^10^

**Fig 3:**
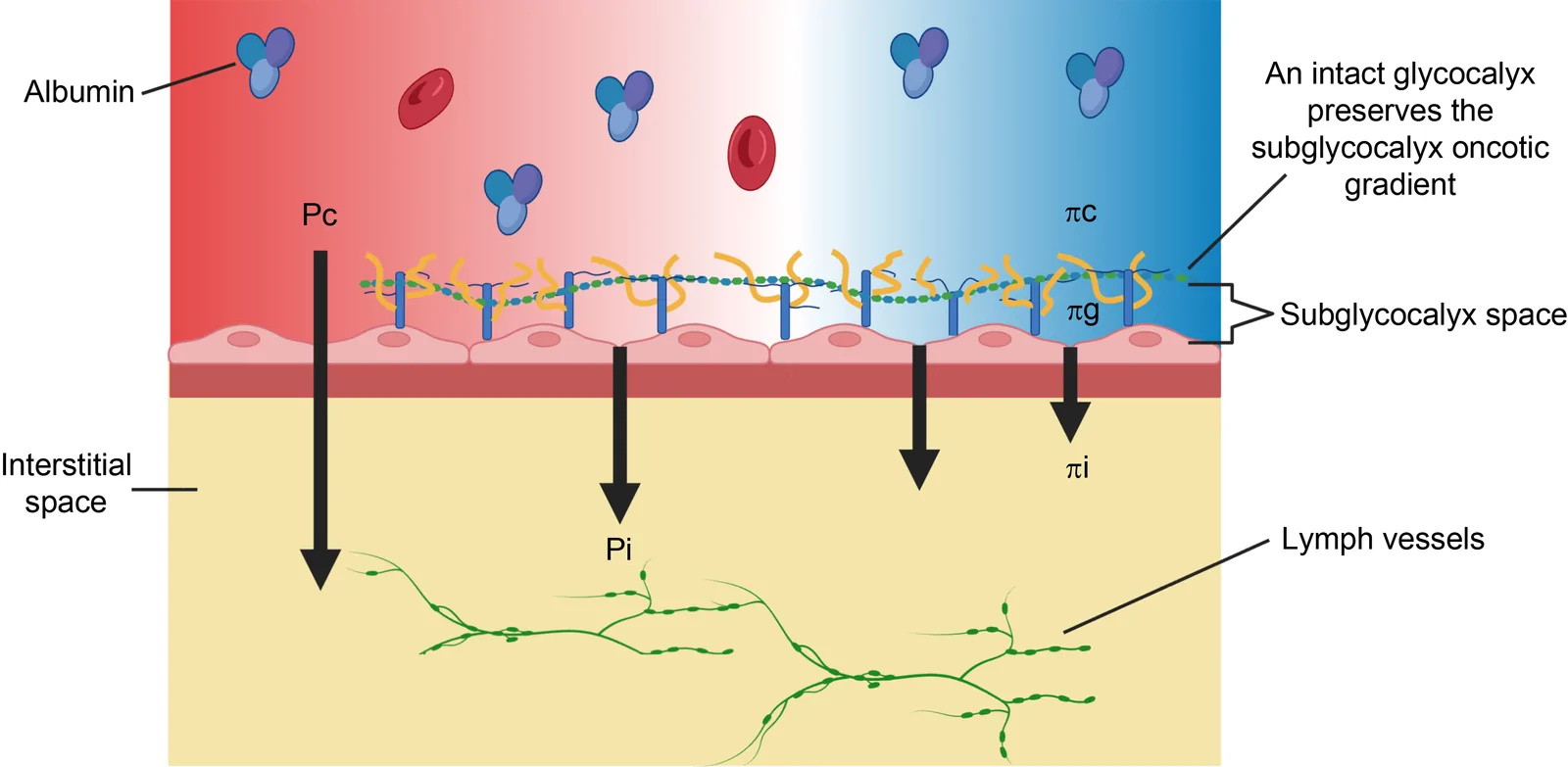
Revised Starling principle. Capillary hydrostatic pressure (Pc) is the principal outward force for filtration and is opposed by interstitial hydrostatic pressure (Pi) and the oncotic gradient between capillary plasma (πc) and the protein-poor subglycocalyx space (πg). Interstitial oncotic pressure (πi) is relatively high. An intact endothelial glycocalyx preserves a low πg, thereby limiting outward fluid filtration. Filtered fluid is returned predominantly via the lymphatic system.

The revised Starling equation therefore describes net transvascular fluid movement as:

Jv = Kf ([Pc − Pi] − σ[πc − πg])

where πg is the subglycocalyx oncotic pressure. Therefore, an oncotic gradient develops from the low protein concentration of the subglycocalyx space to intravascular space, which opposes outward fluid filtration (Fig. 3).

The endothelial glycocalyx is damaged in inflammatory states such as sepsis by sheddases, enzymes that cleave key components of the glycocalyx and are activated by inflammatory cytokines. A multitude of studies have demonstrated that markers of glycocalyx breakdown, such as syndecan-1, hyaluronan and heparan sulphate, are associated with the presence and severity of sepsis, and adverse outcomes.^12^ Glycocalyx damage compromises the integrity of the normally near protein-free subglycocalyx space, which elevates the subglycocalyx colloid oncotic pressure and predisposes to intravascular fluid loss.

## Physiological rationale for i.v. fluid therapy

Despite the ubiquitous use of i.v. fluids in sepsis, no major randomised controlled trial tested their efficacy until 2001, when Rivers and colleagues determined that an early goal-directed therapy approach involving i.v. fluids, vasopressor or vasodilator drugs, inotropic drugs and red blood cell transfusions was associated with an improvement in mortality.^13^ However, because of the multifaceted nature of the intervention, it is difficult to delineate the effects of fluids alone, and three subsequent large randomised controlled trials refuted the role of protocolised resuscitation in sepsis.^14^

### Frank-Starling principle

**Fig 4:**
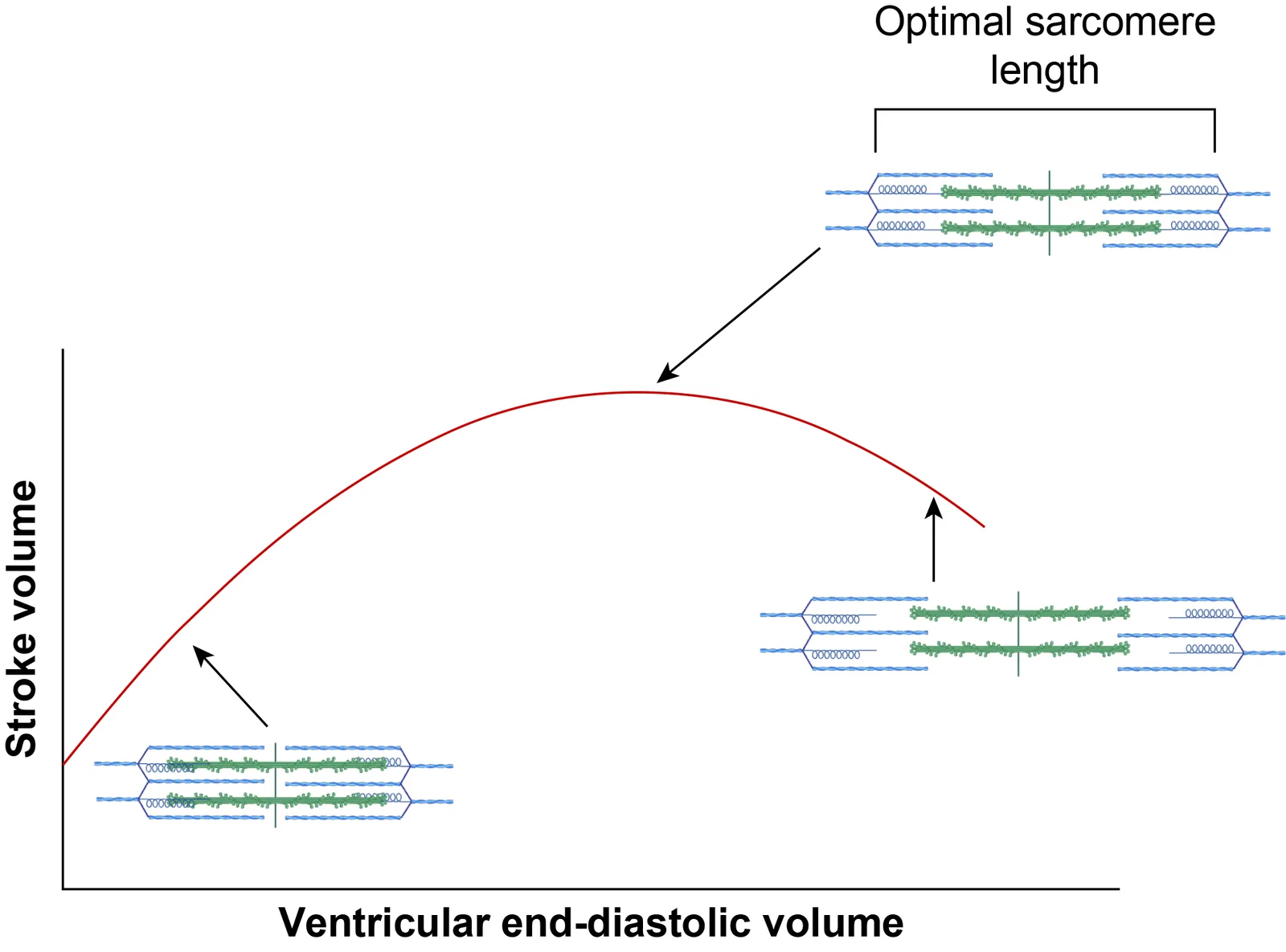
Effect of sarcomere length on stroke volume. Stroke volume increases with increasing preload until an optimal sarcomere length is reached. At shorter or excessive sarcomere lengths, tension generation and contractile performance are reduced.

The primary goal of fluid bolus therapy is to improve cardiac preload to an optimal point, which, according to the Frank-Starling principle, optimises stroke volume and thus cardiac output and oxygen delivery. The Frank-Starling principle describes the relationship between preload, measured as left ventricular end-diastolic volume, and cardiac performance, measured as stroke volume. There is an optimal length of cardiac sarcomeres at which tension in the myocardial muscle fibres is greatest, resulting in maximal contraction and cardiac performance (Fig. 4). However, if the cardiac sarcomeres are too close or too far apart compared with the optimal length, there will be a reduction in tension and contractility. Sarcomere length is directly related to preload, which reflects the stretch applied to individual cardiac cells at the end of diastole and is closely related to ventricular filling. The important question for clinicians is whether an increase in preload will augment stroke volume, that is, whether the patient is ‘fluid responsive’.

### Stressed blood volume

**Fig 5:**
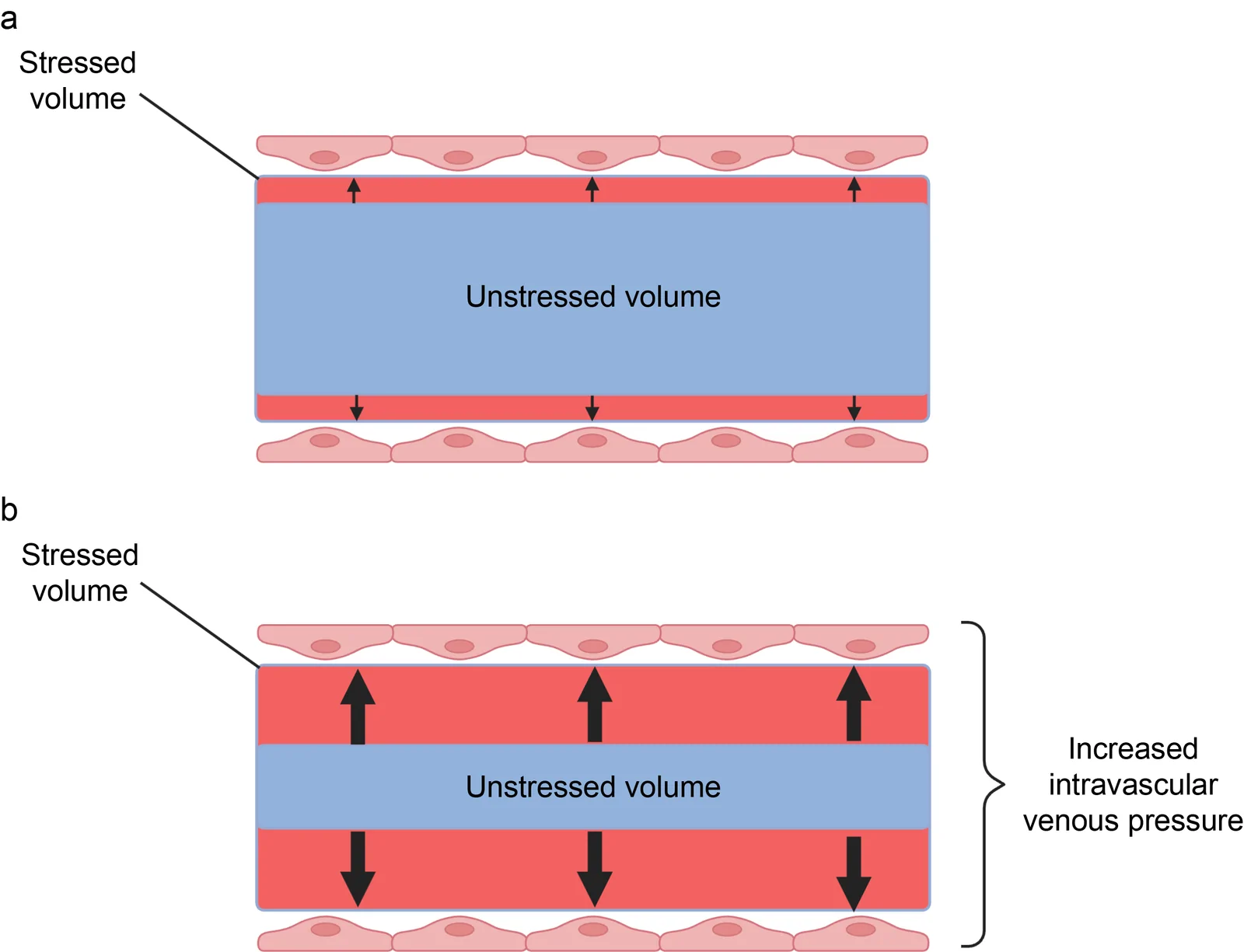
Stressed and unstressed blood volume. a) In the compliant venous system, most blood volume is unstressed and produces little increase in intravascular pressure. b) An increase in stressed volume increases venous pressure and thereby enhances venous return.

Venous return is a critical determinant of cardiac output and is generated by the pressure gradient between the venous system and the right atrium. Myocardial contractility and heart rhythm are also important determinants of cardiac output. As a result of the compliant nature of the venous system, approximately 70% of total blood volume is located within veins. The venous system can be separated into two theoretical compartments, the unstressed and stressed volumes. The unstressed blood volume refers to the volume that fills the venous system before there is any significant rise in intravascular pressure, whereas the stressed blood volume refers to the volume that exerts stretch on the venous vessel walls and causes intravascular pressure to increase (Fig. 5a and 5b).^15^ As such, the stressed blood volume is a major determinant of venous pressure and, therefore, venous return.

In sepsis-induced vasoplegia, the increase in compliance of the venous system causes a shift in the amount of blood in the stressed volume to the unstressed volume, which in turn will reduce venous pressure and venous return. The aim of i.v. fluid therapy in sepsis is to increase the proportion of the stressed blood volume and, in so doing, augment venous pressure and venous return to the right atrium, increasing stroke volume and thus oxygen delivery to tissues. Noradrenaline (norepinephrine) and other vasopressor drugs may have similar effects on venous return by reducing venous capacitance and increasing stressed blood volume, in addition to effects on arterial tone.

Despite a sound physiological rationale, the observed benefits of i.v. fluid challenges are transient. In patients who are critically ill with circulatory shock, fluid resuscitation temporarily improves cardiac index; however, this effect is often short-lived, with values returning towards baseline within 1 h.^16^

## Current fluid resuscitation practice

The FENICE study collected data on fluid challenges in intensive care.^17^ FENICE revealed significant variability in the type, rate and volume of fluid used. Moreover, assessment of fluid responsiveness was not routinely employed and, concerningly, half of the patients with an initial negative response to a bolus of i.v. fluid progressed to receive further fluid challenges.

The Surviving Sepsis Campaign recommends giving at least 30 ml kg^−1^ i.v. crystalloid within the first 3 h of resuscitation in patients with sepsis-induced hypoperfusion or septic shock.^18^ These guidelines advocate the use of dynamic measures such as stroke volume variation or pulse pressure variation to guide resuscitation with fluids.

Recent European Society of Intensive Care Medicine guidelines for treatment with fluids are broadly concordant with this approach. In sepsis, they suggest giving crystalloids rather than albumin, and balanced crystalloids rather than isotonic saline, to expand intravascular volume, and suggest up to 30 ml kg^-1^ i.v. crystalloid in the initial phase with frequent reassessment and subsequent individualisation of therapy.^19^^,^^20^

The ANDROMEDA-SHOCK 2 randomised controlled trial compared a strategy of peripheral perfusion-targeted resuscitation *vs* lactate concentration-targeted resuscitation in patients who are critically ill with septic shock.^21^ A peripheral perfusion-guided strategy resulted in less resuscitation fluid being given and less organ dysfunction at 72 h, although 28-day mortality did not differ significantly between groups. More recently, the ANDROMEDA-SHOCK 2 trial extended this concept by testing a personalised haemodynamic resuscitation protocol targeting capillary refill time against usual care in early septic shock. The intervention reduced resuscitation fluid volume and improved a hierarchical composite outcome of death, duration of vital support, and length of hospital stay at 28 days. These findings further support an individualised physiology-based approach to early resuscitation.^22^

The Surviving Sepsis Campaign guidelines suggest using balanced crystalloids instead of saline 0.9% for resuscitation.^18^ Historically, saline 0.9% solution was the preferred choice of crystalloid; however, concerns relating to adverse effects, including hyperchloraemic metabolic acidosis, potentiated cytokine secretion, and concern about renal injury, have led to increased use of chloride-restrictive solutions, known as balanced solutions.

## Evidence of harm with i.v. fluid therapy

An obvious complication of giving excessive fluids is the development of fluid overload. While poorly defined, fluid overload generally implies a degree of fluid accumulation which manifests clinically as peripheral or pulmonary oedema. An increase in body weight after admission of >5% is the most widely used definition for fluid overload in critical care.^23^

### Observational studies

A large body of evidence shows that giving fluids and the accumulation of a positive fluid balance are associated with adverse outcomes, including mortality in patients who are critically ill. For example, a retrospective analysis of patients recruited to the Vasopressin in Septic Shock Trial (VASST) study identified that a more positive fluid balance, both early in resuscitation and over 4 days, was associated with increased mortality even after correction for illness severity.^24^ Studies have also shown that the degree of fluid overload is independently associated with 28-day mortality in patients who are critically ill with sepsis-induced acute kidney injury (AKI).^25^ However, it is important to remember that observational data cannot distinguish whether fluid overload directly contributes to mortality in patients who are critically ill with septic shock or simply serves as a surrogate marker of greater illness severity.

### Randomised controlled studies

The landmark Fluid Expansion As Supportive Therapy (FEAST) trial investigated the effects of giving boluses of fluids in over 3,000 children presenting with severe febrile illness complicated by impaired consciousness and/or respiratory distress, and impaired perfusion.^26^ The investigators compared the effects of giving a bolus of albumin 5% with a bolus of saline 0.9% and with no fluid bolus. The FEAST study identified significantly increased 48-h mortality in children receiving boluses of fluid: 10.6% in the albumin group compared with 10.5% in the saline group and 7.3% in the control group. The increased mortality in the groups receiving fluids was not attributable to the classical complications associated with excessive fluids, such as pulmonary oedema or raised intracranial pressure, but rather to cardiovascular collapse.^27^

Subsequent to the FEAST trial, Andrews and colleagues investigated a protocolised strategy involving aggressive therapy with fluids, vasopressors, and blood transfusions for resuscitating adults with septic shock.^28^ Patients in the intervention arm received rapid therapy with fluids and a median of 3.5 L within 6 h of hospital admission, compared with patients in the usual care group, who received a median of 2 L fluids. Mortality was significantly increased in patients receiving protocol-guided care (48.1%) compared with the control group (33%).

## Mechanisms of harm with i.v. fluid therapy

Emerging evidence suggests that treatment with i.v. fluids induces glycocalyceal injury, thought to be mediated by shear stress.^29^ In sepsis, where glycocalyceal injury is a prominent feature, the rapid infusion of i.v. fluids is hypothesised to exacerbate glycocalyx injury and thus capillary leak. In a retrospective analysis of patients with sepsis, the volume of i.v. fluids given was independently associated with the degree of degradation of the glycocalyx.^30^ The effects of resuscitation with fluids have been investigated in an ovine endotoxaemia model of sepsis, which compared giving fluids with vasopressor drugs against using vasopressor drugs alone.^31^ Resuscitation with fluids resulted in a paradoxical increase in the need for vasopressor drugs, with an increased rate of glycocalyx shedding, and failed to improve end-organ oxygenation.

### Atrial natriuretic peptide

Harmful effects of i.v. fluids in resuscitation may also be mediated by the release of atrial natriuretic peptide (ANP) from the cardiac atria in response to atrial wall stretch. ANP may act as an endothelial glycocalyx sheddase, and hypervolaemia may increase its release, which subsequently mediates shedding of the endothelial glycocalyx.^32^

### Interstitial fluid accumulation and venous congestion

Excessive interstitial fluid may cause mechanical extrinsic compression of capillaries and lymphatics, which may further worsen oxygen delivery and interstitial drainage, respectively. Moreover, the role of venous congestion in the development of organ failure is worth specific consideration. Studies have shown that, in patients with sepsis, an increased central venous pressure (CVP) is associated with an increased risk of renal injury.^33^ Mechanistically, an increase in CVP will cause an increase in renal venous pressure, resulting in higher renal interstitial and intratubular pressures, which will ultimately reduce the ultrafiltration pressure gradient. Similarly, a higher renal venous pressure may attenuate renal blood flow via a reduction in the transrenal arteriovenous pressure gradient. Reduced renal ultrafiltration and renal blood flow will inevitably result in a decreased urine output, which often erroneously prompts the clinician to give further i.v. fluids. However, this may only serve to further increase venous pressure and worsen congestion. In patients with advanced decompensated heart failure, increased CVP has emerged as a stronger predictor of deteriorating renal function than cardiac index, thereby highlighting the central role of venous congestion in the pathogenesis of cardiorenal syndrome.^34^ This concept is also relevant in SCM, where any putative haemodynamic benefit from expanding intravascular volume must be balanced against the risk of worsening venous congestion, pulmonary oedema and organ dysfunction.

### Fluid-restrictive strategies

The combination of retrospective and randomised controlled trial evidence pointing towards harm from i.v. fluids in sepsis has led to the investigation of the safety and efficacy of alternative, fluid-restrictive strategies.

### CLASSIC trial

The CLASSIC trial compared a fluid-restrictive strategy with standard fluid therapy in 1,554 patients with septic shock.^35^ Participants in the restrictive fluid group could receive i.v. fluid in cases of severe hypoperfusion, ongoing fluid losses, or to maintain hydration and correct electrolyte abnormalities if the enteral route was contraindicated. During the trial period, patients in the restrictive fluid group received a median of 1,798 ml of fluid (interquartile range [IQR], 500–4,366), whereas patients in the standard care group received a median of 3,811 ml of fluid (IQR, 1,861–6,762). The CLASSIC trial failed to demonstrate efficacy for one approach over the other, with similar 90-day mortality between the groups: 42.3% in the restrictive fluid group *vs* 42.1% in the standard fluid group.^35^ Importantly, both approaches were safe, with no differences in adverse outcomes between groups. Of note, participants in both arms received a significant volume of i.v. fluids before trial recruitment (median 3 L in the standard care groups compared with 3.2 L in the restrictive group), which may have nullified any potential benefits of a restrictive fluid regimen.

### CLOVERS trial

The CLOVERS trial randomised 1,563 patients with sepsis-induced hypotension to either a restricted or liberal fluids strategy for a 24-h period.^36^ Patients in the restricted fluids group received limited fluids and preferentially received vasopressors to provide haemodynamic support. Patients in the restricted fluids group received less fluid than those in the liberal fluids group (difference of medians, −2,134 ml). The CLOVERS trial revealed similar rates of death before discharge home at 90 days between groups: 14.0% in the restricted fluids group and 14.9% in the liberal fluids group.

## Evidence-based management of sepsis-induced hypotension

The SSC guideline recommendation of giving a bolus of 30 ml kg^−1^ fluids for sepsis-induced hypotension is based on the average volume given to patients before randomisation in clinical trials. It will be challenging to move away from this fixed-volume resuscitation recommendation as a starting point without robust evidence directly comparing a 30 ml kg^−1^ strategy to less or more fluid based on clinical context. However, as clinicians, we have a number of tools and data at our disposal with which to individualise therapy.

It is likely that certain groups of patients with sepsis will benefit from more restrictive or more liberal approaches. These groups of patients need to be characterised such that an individualised approach can be adopted. The failure of various randomised controlled trials in sepsis to produce definitive conclusions is likely to be a result of the heterogenous nature of sepsis in clinical practice. Investigators have used various ultrasound-based techniques to characterise patients physiologically, aiming to distinguish those who are fluid responsive, meaning they can tolerate fluid expansion without adverse effects, from those who are intolerant of fluids.^37^

**Fig 6:**
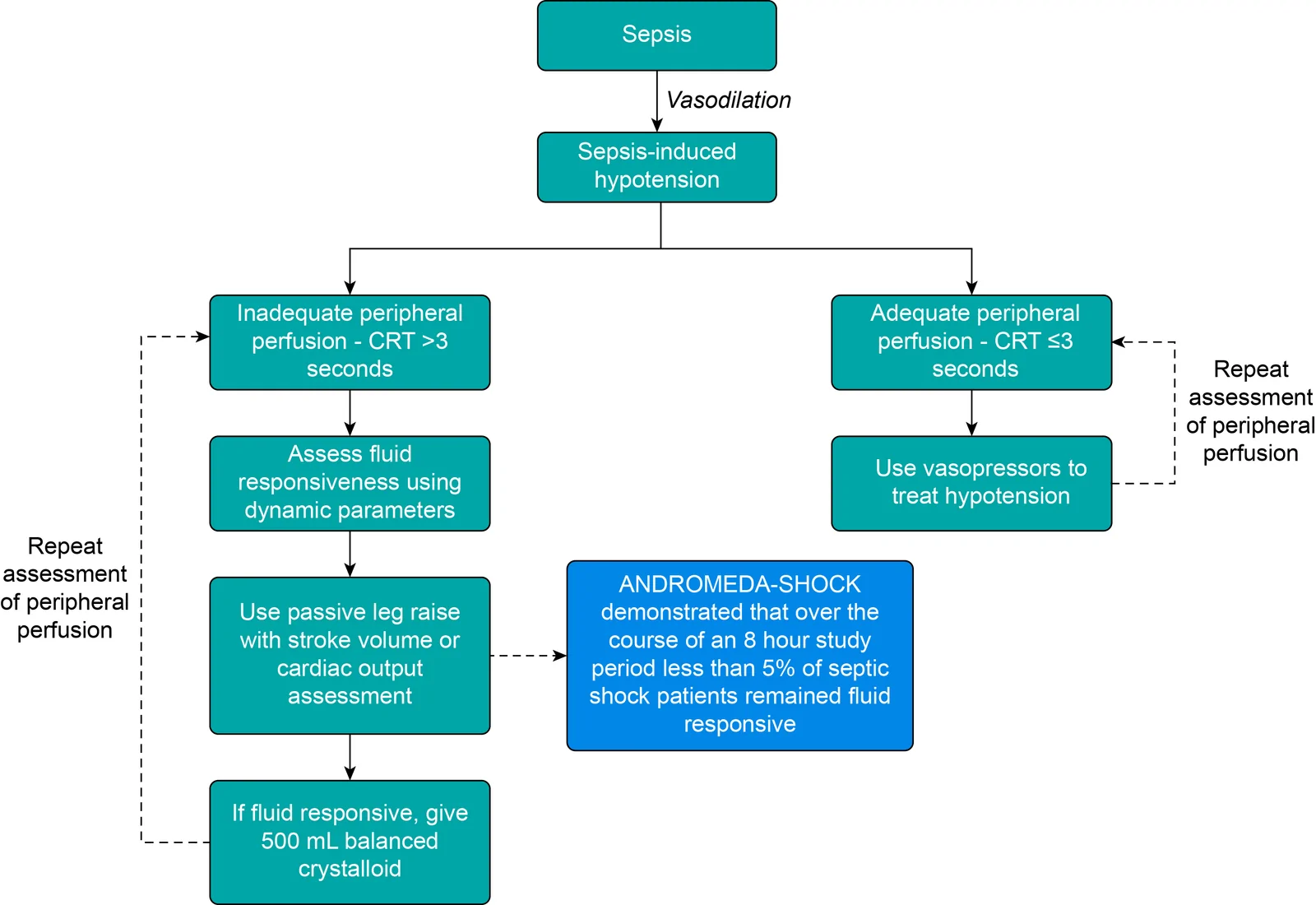
Algorithm for fluid therapy in sepsis-induced hypotension based on peripheral perfusion, using capillary refill time (CRT) >3 s to define inadequate perfusion.

The predominant problem in sepsis-induced hypotension is vasoplegia rather than absolute hypovolaemia. The peripheral perfusion of patients with sepsis-induced hypotension should be examined and, if inadequate, that is, capillary refill time >3 s or extensive skin mottling, fluid responsiveness should be assessed using dynamic measures (Fig. 6). Data from the ANDROMEDA-SHOCK trial demonstrated that the vast majority of patients with sepsis admitted to critical care rapidly become fluid unresponsive.^21^ The use of dynamic measures of fluid responsiveness, such as the passive leg raise manoeuvre, is to predict whether stroke volume will increase in response to a bolus of fluids, which may or may not in turn result in improved perfusion. Predictors of fluid responsiveness are not indicative of hypovolaemia, and even when present, do not comprise an indication for giving i.v. fluids in themselves.

An important corollary is that in patients who are adequately perfused, with acceptable capillary refill time (≤3 s), absence of extensive skin mottling, and other markers, there is little basis for giving i.v. fluids in an attempt to improve circulation; if needed, vasopressors should instead be used to treat pathological vasodilation.

The CLASSIC trial demonstrated that it was safe to tolerate a degree of hypoperfusion in patients with septic shock.^35^ Patients randomised to receive a regimen of restricted fluids only received additional fluids in cases of severe hypoperfusion, that is, a serum lactate concentration of at least 4 mmol L^−1^ and mottling beyond the edge of the kneecap (mottling score >2). The mottling scoring system is based on the presence and extension of mottling on the legs (Table 1).^38^ The mottling score has been demonstrated to have a high degree of interobserver agreement and is a strong predictor of mortality in septic shock.

**Table 1:** Mottling score.

Mottling score	Description
Score 0	No mottling
Score 1	Coin-sized and localised to the centre of the knee
Score 2	Mottling does not exceed the superior edge of the kneecap
Score 3	Mottling does not exceed the middle thigh
Score 4	Mottling does not exceed beyond the fold of the groin
Score 5	Mottling exceeds beyond the fold of the groin

Fluid overload is common after admission to the intensive care unit. Studies have shown that diluents for medications and maintenance fluids are the largest contributors to fluid input in patients who are critically ill.^39^ Therefore, in order to prevent the development of fluid overload, maintenance fluids should almost always be discontinued. Patients who are critically ill should be assessed on a regular basis for the development of interstitial oedema and, if associated with organ dysfunction, fluid deresuscitation should be considered, provided there are no contraindications. Recent European Society of Intensive Care Medicine guidance on the de-escalation phase supports fluid de-escalation after the acute phase and protocolised diuretic-based fluid removal, while advising against routine ultrafiltration or extracorporeal fluid removal unless another indication for renal replacement therapy exists.^40^ However, further work is needed to determine whether deresuscitation improves outcomes and, if so, the optimal timing, rate, and method of fluid removal. This would be assisted by the identification of sensitive and accurate markers of intravascular and interstitial volume status. Although ultrafiltration may allow cautious and controlled removal of fluids in selected patients, its routine use for deresuscitation cannot currently be recommended.

## Fluids in other intensive care unit conditions

### Pancreatitis

Pancreatitis is recognised as a common cause of sterile inflammation capable of inducing a systemic inflammatory response. It has been demonstrated that a strategy of rapid volume expansion in patients with severe pancreatitis is associated with higher Acute Physiology and Chronic Health Evaluation II scores, rates of mechanical ventilation, incidences of abdominal compartment syndrome and sepsis, and reduced survival. The WATERFALL randomised trial compared aggressive with moderate fluid resuscitation in patients with acute pancreatitis. The trial was halted early because aggressive resuscitation with fluids increased fluid overload (20.5% compared with 6.3%) without improving clinical outcomes.^41^

### Acute respiratory distress syndrome

The inflammatory-mediated barrier breakdown in acute respiratory distress syndrome (ARDS) overlaps with sepsis. The Fluid and Catheter Treatment Trial (FACTT) was a randomised study that compared conservative fluid therapy with liberal fluid therapy in 1,000 patients with ARDS.^42^ Although there was no statistically significant difference in the primary outcome of 60-day mortality, a conservative fluid strategy resulted in an improved oxygenation index, Murray lung injury score, and a reduced duration of mechanical ventilation and length of intensive care unit stay. A post-hoc analysis of FACTT investigated the incidence of AKI between the conservative fluid therapy and liberal fluid therapy arms.^43^ Following adjustment for fluid balance, the incidence of AKI was higher in those managed with the liberal fluid protocol (66% *vs* 58%, *p* = 0.007). ARDS is a heterogeneous syndrome, with hyperinflammatory and hypoinflammatory subphenotypes. The hyperinflammatory subphenotype is characterised by higher concentrations of pro-inflammatory cytokines and endothelial injury biomarkers. Latent class analysis of FACTT identified these subphenotypes and suggested heterogeneity of treatment effect, with fluid strategy affecting 90-day mortality differently between subphenotypes.^44^ The mortality rate in the hypoinflammatory subphenotype was 26% with a liberal fluid approach compared with 18% with a conservative fluid approach, while in the hyperinflammatory subphenotype, mortality was 40% with the liberal fluid strategy *vs* 50% with the conservative fluid strategy. This analysis is supportive of tailored fluid therapies depending on the ARDS subphenotype. However, a follow-up study of FACTT survivors identified an association between the conservative fluid strategy and long-term cognitive impairment. This exploratory finding requires confirmation, but highlights that short-term physiological benefits may not necessarily translate into superior long-term outcomes.^45^

## Conclusions

Despite a physiological rationale for giving i.v. fluids in sepsis-induced hypotension, associated improvements in haemodynamics are often minimal and transient. The liberal, aggressive infusion of fluids will often lead to tissue oedema and circulatory overload, with a substantial body of evidence highlighting the harm of excessive i.v. fluids in sepsis. Effective strategies to guide the use of fluids are therefore crucial. Emerging evidence supports an approach based on peripheral perfusion, offering a more individualised strategy to therapy and potentially reducing the risks of fluid overload. Regardless of the strategy, regular reassessment is vital to identify signs of fluid overload and adjust treatment, ensuring a balance between adequate perfusion and avoiding overload-related complications.

## MCQs

The associated MCQs (to support CME/CPD activity) will be accessible at www.bjaed.org/cme/home by subscribers to BJA Education.

## Declaration of interests

The authors declare that they have no conflicts of interest.
